# Enhanced *Salmonella* mortality in naturally resistant, immunocompetent male mice is associated with an altered systemic immune response

**DOI:** 10.1128/iai.00028-26

**Published:** 2026-03-11

**Authors:** Aliyah N. Bennett, Allysa L. Cole, John S. Gunn

**Affiliations:** 1 Center for Microbe & Immunity Research, Abigail Wexner Research Institute at Nationwide Children’s Hospital 51711, Columbus, Ohio, USA; 2 Infectious Diseases Institute, The Ohio State University, Columbus, Ohio, USA; 3 Department of Veterinary Biosciences, The Ohio State University 198563 , Columbus, Ohio, USA; 4 Department of Pediatrics, College of Medicine, The Ohio State University 12305, Columbus, Ohio, USA; University of California Davis, Davis, California, USA

**Keywords:** sexual dimorphism, bioluminescent, *in vivo *imaging, *Salmonella*, inflammation, cytokines, cholecystitis, gallstones

## Abstract

While typhoid fever affects both sexes at an equal rate, males are at a higher risk for intestinal perforation, which increases mortality. The mechanisms behind the increased morbidity of typhoid fever in human males remain an important but understudied question. Using a 129X1/SvJ (NRAMP^+/+^ [*SLC11A1*]) murine model of typhoid chronic infection, we determined that males in this model exhibit increased bacterial burden and mortality in comparison to females (median survival 7 vs 11 days post-infection, respectively). This decreased survival in males was influenced by the diet preceding infection as male mice fed a lithogenic diet, to induce gallstones important to chronic infection, or a normal diet had a lower median survival time, although no difference in the overall probability of survival was observed. We also explored how the systemic immune response may contribute to increased mortality by comparing serum cytokine levels between males and females. Males had higher overall levels of pro-inflammatory cytokines and IL-10 and lower levels of IL-27, which are known to inhibit the protective responses to *Salmonella* infection. Together, we present the first report that the 129X1/SvJ murine model of *Salmonella* infection responds to infection in a sex-dependent manner, characterized by maladjusted systemic cytokine production and increased bacterial burden in males.

## INTRODUCTION

*Salmonella enterica* subspecies *enterica* serovar Typhi (*S*. Typhi) is the causative agent of typhoid fever in humans (1, 2). Acute typhoid fever is characterized by fever, malaise, rash, and abdominal pain (1, 2). Chronic typhoid fever is mediated by *S*. Typhi forming biofilms on gallstones and in the biliary tree (3). While mortality and transmission rates have declined in areas with adequate access to safe drinking water and sanitation, typhoid fever remains endemic in low- to middle-income countries with an incidence of 9 million cases and 110,000 deaths per year (1, 2, 4). Acute typhoid fever is treatable with antibiotics; however, treatment may be complicated by meningitis, sepsis, or intestinal perforation (2). In addition, there are increasing reports of *S*. Typhi with resistance to fluoroquinolones, third-generation cephalosporins, and macrolides (5, 6).

Notably, clinical studies have observed that males are more likely to suffer from intestinal perforation (7–10). Studies in Africa suggest that intestinal perforation is a significant contributor to mortality, with estimates that one in five patients with typhoidal intestinal perforation died as a result (10). Sexual dimorphism in response to specific infections is well-established in many organ systems including the gastrointestinal tract, respiratory tract, and urogenital tract (7, 11–14). However, despite suggestive clinical evidence of sexual dimorphism contributing to different outcomes in typhoid infection, the mechanisms of action and pathology that underlie this phenomenon remain understudied.

*S*. Typhi is a human-restricted pathogen, limiting the ability to study its pathogenesis *in vivo* in immunocompetent mice. *Salmonella* Typhimurium, a serovar closely related to Typhi, is commonly employed to model acute gastroenteritis in susceptible mouse strains, as well as a typhoid-like illness in resistant mouse strains. A benefit of using mouse strains that are less susceptible to *Salmonella* infection, like the 129X1/SvJ strain, is that they can be used to model both an acute and chronic, typhoid-like illness, rather than the gastroenteritis or lethal systemic infection that occurs in sensitive mice. Regardless of the route of infection (i.e., oral gavage, intravenous, and intraperitoneal injection), the inflammatory response begins with phagocytosis of *Salmonella* by dendritic cells and macrophages, which then traffic live bacteria in *Salmonella-*containing vesicles (SCV) to gut associated lymphoid tissue and more distant sites such as the liver and spleen (15–18). Thus, the host response of less susceptible mouse strains is characterized by both systemic and local inflammatory responses (19–25). However, most studies using *Salmonella*-infected 129X1/SvJ mice to study the host response use cohorts of the same sex (19, 20, 26–28) or do not specify the sex of the mice used (29). Of studies that do use both sexes, many do not disaggregate data between the sexes or perform formal comparisons between the two (30, 31). Some studies have explored the role of female sex hormones on mortality after *Salmonella* infection; however, they used sensitive mice that are not ideal for studying typhoidal illness (32, 33). Moreover, chronic typhoidal carriage is arguably best modeled in resistant mice with cholelithiasis, replicating the gallstone colonization important to human disease. To date, studies using resistant mouse strains demonstrating a sex-based difference in outcomes of *Salmonella* infection have not incorporated cholelithiasis into their models (34).

Thus, we are limited in our ability to understand how biological sex may contribute to the host response following *Salmonella* infection, especially in the case of chronic infections. We aim to address this question by comparing the response to infection in male and female mice in a murine model of chronic typhoid infection to determine if this model also presents with sex differences in disease outcomes, like those observed in human disease. We also explore the differences in the systemic immune response between males and females and their relationship to infection outcomes.

## MATERIALS AND METHODS

### Bacterial strains

Bacterial strains used in this study include *Salmonella* Typhimurium 14028 (WT) and the previously described *S*. Typhimurium with a chromosomal *lux* operon (14028lux) (21). WT and 14028lux were cultured in Luria-Bertani (LB) liquid media and LB with 40 µg/mL kanamycin (LB+Kan), respectively.

### Mouse infection

A total of 210, 8–10 week old 129X1/SvJ mice (The Jackson Laboratory) were used in this study. At the beginning of the experiments, mice were either fed a lithogenic diet (LD) (conventional mouse chow supplemented with 1% cholesterol and 0.5% cholic acid) (Envigo, Rodent Diet 7012, 1% Chol., 0.5% C.A. [TD.140,673]) for 6–8 weeks in order to induce gallstone formation or conventional mouse chow (normal diet [ND]) (Envigo, Rodent Diet 7012). All mice were maintained on an ND after the initial diet period and were allowed at least a 1-week rest period on n ND prior to infection. Methods for mouse infection were performed as previously described (21). In brief, mice were intraperitoneally (i.p.) injected with 200 µL (8.2 × 10^2^–5.8 × 10^3^ colony-forming units [CFUs]) of WT, 14028lux, or an equal volume of sterile phosphate-buffered saline (PBS). The bacterial inoculum was validated by plating each inoculum to LB agar or LB+Kan agar and then enumerating the CFUs present after an overnight incubation at 37°C. Mice were monitored twice daily for clinical, behavioral, physiological, or anatomical signs indicating disease progression to humane endpoints. Any mice reaching the moribund status before the predetermined end of the experiment were immediately humanely euthanized. Humane euthanasia was performed in accordance with the institutional IACUC policy and included a primary method of euthanasia, followed by a secondary physical method for confirmation of death. The primary methods of euthanasia used in this study are either CO_2_ asphyxiation or cervical dislocation after inhaled isoflurane anesthesia. Confirmation of death was achieved through removal of a vital organ or exsanguination.

### Bioluminescent imaging

A total of 147 male and female mice were fed an LD for 8 weeks followed by 1 week on an ND. A total of 143 mice were injected i.p with 1.14 × 10^3^ CFUs of 14028lux in 200 µL sterile PBS. Four mice were i.p injected with an equal volume of sterile PBS. Mice were imaged using the Xenogen Spectrum CT, as previously described (21). In brief, mice were anesthetized with 1% isoflurane during imaging and then returned to their cages to recover in between imaging sessions. Photographic and bioluminescent images were acquired on “auto” in order to optimize the image quality and analyzed using Living Image software. Luminescence was quantified in units of radiance (p/sec/cm^2^/sr). Multiple images were combined into one sequence and normalized to the same radiance scale. Mice injected with PBS were included as a control in order to subtract background auto-luminescence. Mice were imaged every 2–5 days for 64 days. At the conclusion of the experiment, mice were euthanized, and the whole gallbladder and spleen and sections of the liver, spleen, and whole blood were homogenized in sterile PBS using a mechanical homogenizer. Organ homogenates were plated to LB+Kan agar for CFU enumeration, as described above. CFUs were normalized to the mass of tissue and reported as CFU/mg. CFU data visualization and statistical analysis were performed using GraphPad Prism 10.

### Quantification of complete blood count and serum cytokines

Whole blood was collected from the facial vein of individual mice at 4 and 11 days post-infection (dpi) or post-mortem via cardiac puncture at 9, 11, or 21 dpi. Immediately after blood collection, a drop of blood was applied to a glass slide, smeared, and stored until staining. Slides were stained with modified Wright-Giemsa, and a 100-cell differential was performed. Total white blood cell count was calculated by averaging the number of leukocytes in 10 fields, using a 10× eyepiece and 50× oil-immersion lenses.

The remaining collected blood was allowed to clot for at least 30 min at room temperature, before serum isolation. Coagulated blood was centrifuged to separate the serum fraction and then stored at −20°C. Serum IFN-γ, TNF, and IL-10 were measured individually using BD OptEIA ELISA kits (Cat. No. 555138, 555268, and 555252, BD Biosciences) according to the manufacturer’s protocol. Additionally, serum IL-1α, IL-1β, IL-6, IL-10, IL-12p70, IL-17A, IL-23, IL-27, CCL2 (MCP-1), IFN-β, IFN-γ, TNF-α, and GM-CSF were measured simultaneously using the LEGENDplex Mouse Inflammation panel (Cat. No. 740446, Biolegend) according to the manufacturer’s protocol. Bead fluorescence was measured using the Beckman Coulter-Cytoflex using the manufacturer-suggested setup protocol and cytokine concentration calculated using LEGENDplex data analysis software.

### Statistics and reproducibility

Detailed information on statistical analyses, sample sizes, and replicates can be found in the figure legends for each experiment. In brief, statistical analyses were performed using GraphPad Prism 10. For all experiments with multiple statistical analyses on a single data set, distributions are assumed to be normal. The comparison between groups employs statistical tests appropriate for multiple groups and corrects for multiple comparisons using the described statistical hypothesis test.

## RESULTS

### Increased mortality of *Salmonella*-infected male mice

**Fig 1:**
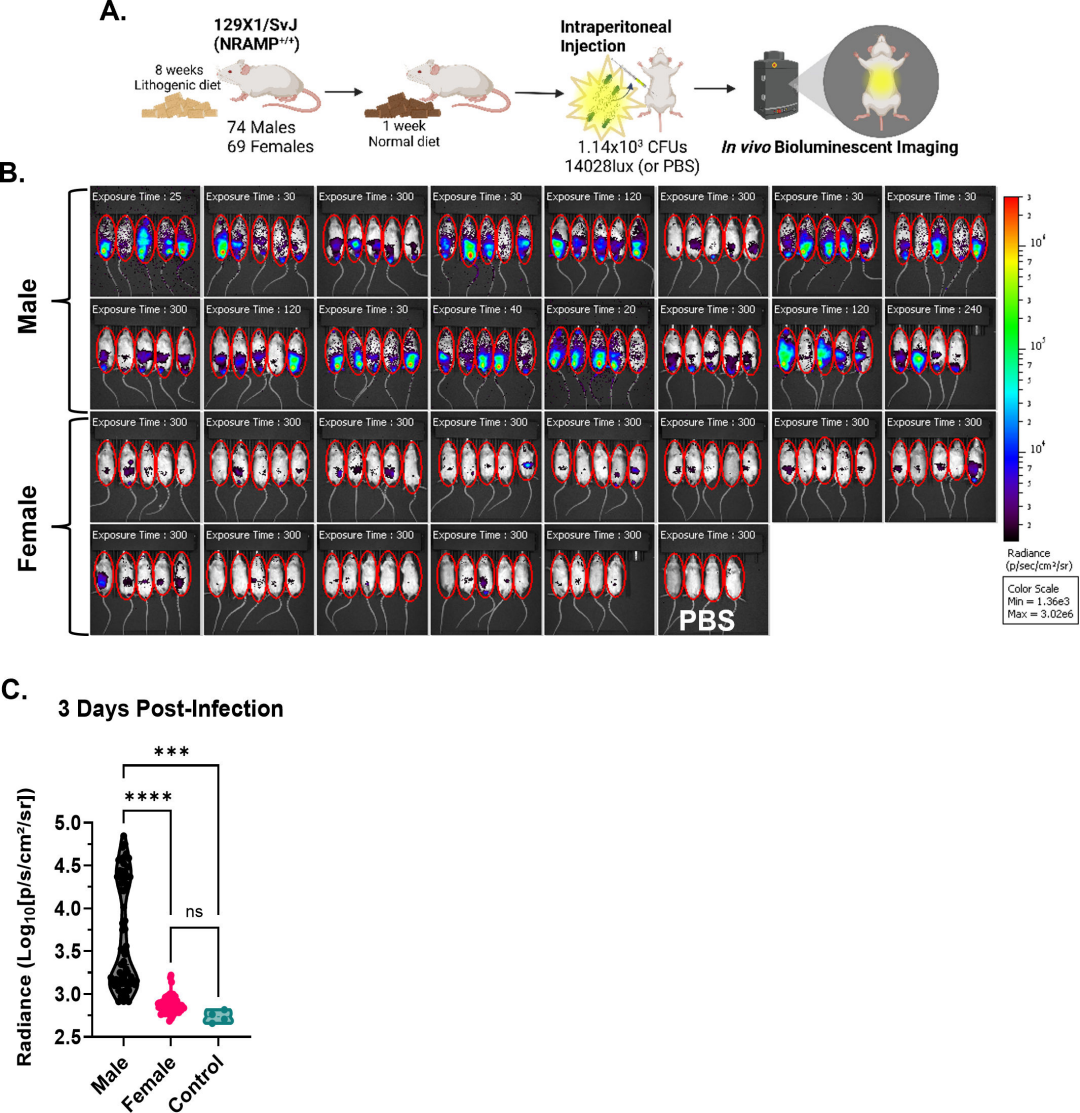
Male mice present with early severe infection. (**A**) Male (*n* = 74) and female (*n* = 69) 129X1/SvJ mice were fed a lithogenic diet and subsequently injected i.p with 1.14 × 10^3^ CFUs of 14028lux. As an auto-luminescence control, 4 female mice were injected with an equal volume of phosphate buffered saline (PBS). Created with Biorender.com. (**B**) At 3 days post-infection, mice were anesthetized and imaged with the Xenogen Spectrum CT. Each image represents an individual cage of mice. Male mice are pictured in the top two rows and female mice in the bottom two rows. Control mice injected with PBS are indicated by the “PBS” label in the image. All images are normalized to the same radiance scale, and exposure time in seconds is indicated in each image. Regions of interest (ROI), in red, were drawn around all mice. (**C**) The average radiance per ROI drawn in **B** was quantified and compared between groups via a one-way ANOVA with Holm-Šídák’s multiple comparisons test. The distribution of data per group is presented in a truncated violin plot, with the dashed line indicating the median, dotted lines indicating quartiles, and symbols representing individual subjects. ns = not significant; ***P < 0.001; ****P < 0.0001.

**Fig 2:**
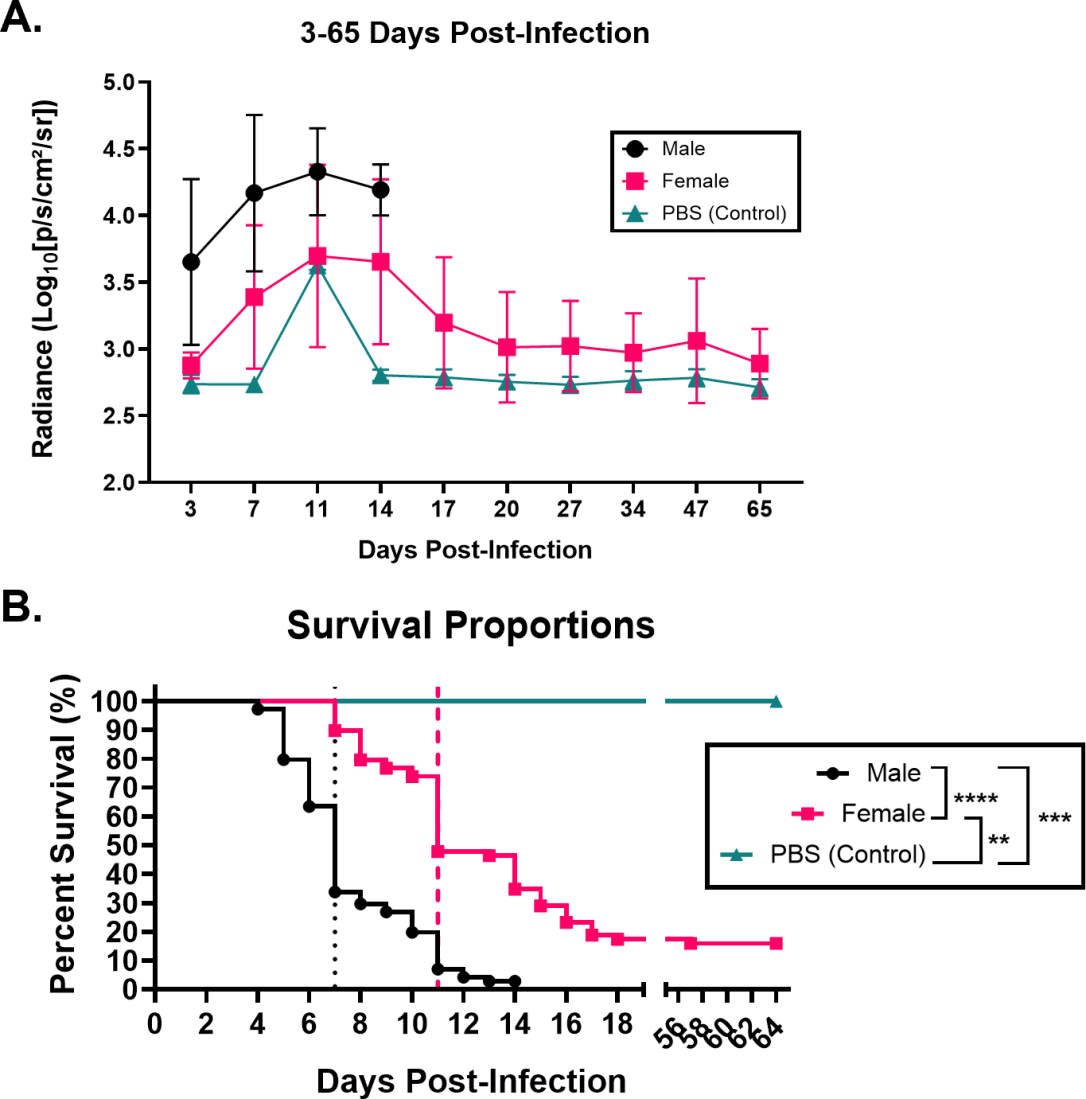
Increased infection severity persists in male mice and is associated with a lower probability of survival than female mice. (**A**) Every 3–13 days post-infection, mice from Fig. 1 were anesthetized and imaged with the Xenogen Spectrum CT. Regions of interest (ROI) were drawn around all mice, and the average radiance per ROI drawn was quantified. The data are represented as the average radiance and standard deviation per group at each time point. (**B**) Probability of survival, median survival time, and difference in survival were calculated using GraphPad Prism 10. The median survival of infected males (7 days post-infection) is indicated by the black dotted line and infected females (11 days post-infection) by the pink dashed line. Survival curves were compared by a log-rank (Mantel-Cox) test, and all comparisons and results are indicated in the figure legend. ns = not significant; **P < 0.01; ***P < 0.001; ****P < 0.0001.

The 129X1/SvJ mice are known to be less susceptible to *Salmonella* infection in part due to the NRAMP1 (*SLC111A1*)^+/+^ allele, and they have been used to model typhoid-like illness in mice (24). However, it has not been established if this mouse model exhibits any sex-dependent differences regarding *Salmonella* infection and chronic carriage. To address this question, we fed 74 male and 69 female 129X1/SvJ mice a lithogenic diet to establish gallstones and intraperitoneally infected these mice with 1.14 × 10^3^ CFUs of 14028lux, a bioluminescent strain of *S*. Typhimurium (Fig. 1A). To monitor the changes in infection, we utilized the Xenogen IVIS system to visualize and measure changes in the luminescent intensity of infected mice (Fig. 2A). Within 3 days, we could observe a marked difference in the luminescent signal between the sexes, with males having a more intense and broadly distributed signal (Fig. 1B). Collectively, males presented with a significantly higher luminescence than females, while the luminescence from female mice was no different from that of controls at 3 days post-infection (Fig. 1C).

By day 7 post-infection, over half of males had died (Fig. 2B), and those that remained had broadly distributed luminescence by comparison to females whose luminescent signals localized to their abdomens (Fig. S1a and d). At 7 and 11 days post-infection, males collectively had significantly higher luminescence than females (Fig. S1a through e). At 14 days post-infection, only two males remained (and were subsequently euthanized due to the moribund status), representing 3% of the initial cohort, by comparison to 38% of females remaining (Fig. S1c and f). A subset of these female mice maintained persistent, chronic infection, as indicated by the presence of luminescence between 20 and 65 days post-infection (Fig. S2). Overall, the survival of males was significantly different from that of females (Fig. 2B), which had not previously been observed in this mouse model. This prompted a deeper investigation of factors that may have contributed to increased mortality in male mice.

Additionally, we considered if this difference in mortality was related to the presence of gallstones by infecting mice fed either a LD or ND for 8 weeks prior to infection (Fig. S3a). We have previously established that the 14028lux isolate has equal fitness to WT (21), so all subsequent experiments were performed with WT 14028. Following the lithogenic diet and one additional week on ND, the mice were infected i.p. with either 5.8 × 10^3^ CFUs of WT STm or an equal volume of sterile PBS (Fig. S3a). During the experiment, the mice were monitored for morbidity, and all remaining mice were euthanized at 9 days post-infection. To compare the bacterial burden, we collected gallbladder, liver, spleen, cecum, and whole blood to enumerate *Salmonella* CFUs in infected mice. We observed that the probability of mortality is independent of the diet prior to infection. Lithogenic diet-fed male mice demonstrated significantly decreased survival probability in comparison to lithogenic diet-fed female mice, and normal diet-fed mice demonstrate a similar trend, although not statistically significant (Fig. S3g).

Of the mice that survived to day 9 post-infection, we quantified bacterial CFUs in the gallbladder, liver, spleen, cecum, and peripheral blood. While all groups had at least 100 CFUs in the gallbladder, liver, spleen, and peripheral blood, supporting that infection was successful, there was no significant difference in the bacterial burden in these organs (Fig. S3b through e). The cecum was an exception to this trend, and we observed significantly decreased cecal bacterial load in ND-fed male mice in comparison to LD-fed male mice and ND-fed female mice (Fig. S3f).

### *Salmonella* infection is associated with sex-specific systemic leukocyte populations

*Salmonella* gains access to the systemic circulation by surviving phagocytic killing within SCVs and then is transported by these phagocytes through systemic circulation to the spleen, liver, and other lymphocytic organs (35–39). This triggers expansion of both innate and adaptive lymphocytic populations to control infection, reflected in increases in white blood cell (WBC) counts in peripheral blood (37–39). Additionally, because we can induce cholelithiasis in these mice that model chronic *Salmonella* infection, we can also observe how the transition from acute infection to chronic infection mediated by *Salmonella* biofilm formation on gallstones (3, 23, 24, 40) affects the immune response.

**Fig 3:**
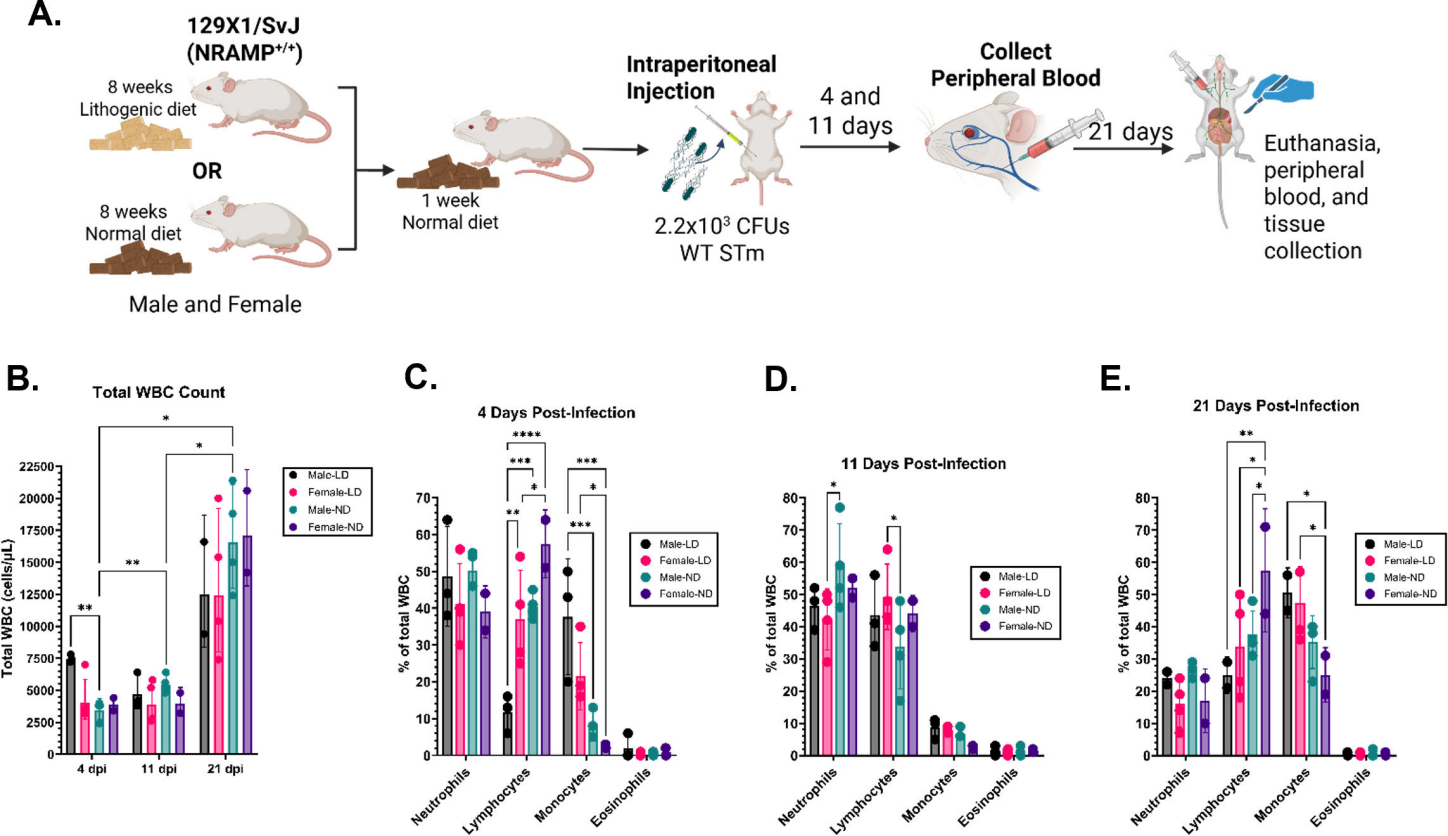
Acute *Salmonella* infection in male mice is associated with higher numbers of peripheral WBCs. (**A**) Male and female mice were fed either lithogenic diet (LD) (male *n* = 3 and female *n* = 4) or normal diet (ND) (male *n* = 4 and female *n* = 2) for 8 weeks followed by 1 week on ND. Mice from each dietary condition were then infected via an intraperitoneal injection with 2.2 × 10^3^ CFUs of WT *S*. Typhimurium. Peripheral blood was collected from individual mice at 4 and 11 days post-infection (dpi) via the facial vein and at 21 dpi via a post-mortem cardiac puncture. Immediately following the blood draw, blood smears were created using glass slides and allowed to dry prior to staining with a Modified Wright-Giemsa stain. Absolute white blood cell (WBC) count and per cell type per µL of blood (cells/µL) was calculated by counting the number in ten microscopic fields per slide at (**B**) 4, 11, and 21 dpi. The graph represents the average and standard deviation of total WBC cells/µL per group, with symbols representing individual subjects. Groups are compared via a mixed-effects analysis with Tukey’s multiple comparisons test. The proportion of neutrophils, lymphocytes, monocytes, and eosinophils, out of the total WBC population, at (**C**) 4, (**D**) 11, and (**E**) 21 dpi was calculated and compared via a two-way ANOVA with Tukey’s multiple comparisons test. Graphs represent the average and standard deviation of each cell type’s % of the total WBCs. *P < 0.05; **P < 0.01; ***P < 0.001; ****P < 0.0001; All other comparisons are not significant.

However, cholelithiasis is associated with a pro-inflammatory state in mice (41). To account for dietary influence on the immune response, we fed male and female mice either LD or ND for 8 weeks, followed by a 1-week period in which all mice were maintained on ND. Given that the infectious dose used in Fig. 1 as well as the infectious dose in Fig. S3 resulted in increased mortality in males, we aimed to give the mice a lower infectious dose so that we might trigger an immune response without causing mortality. Following intraperitoneal infection with 2.2 × 10^3^ CFU of WT 14028, we serially quantified WBC populations in the peripheral blood of infected mice from early, acute infection (4 and 11 days post-infection) to chronic infection (21 days post-infection) (Fig. 3A). Of note, only one LD-fed male mouse became moribund (and was subsequently euthanized on day 19 post-infection) during the experiment shown in Fig. 3.

Early in infection (4 days post-infection), LD-fed male mice had a significantly higher absolute WBC count than ND-fed male mice, as well as a not significant, but higher absolute WBC count than LD-fed female mice (Fig. 3B). Later in infection at 11 and 21 days post-infection, absolute WBC counts normalize between groups (Fig. 3B). All groups have higher WBC counts at 21 days post-infection in comparison to 4 and 11 days post-infection, although this difference is only statistically significant in the ND-fed male mice (Fig. 3B).

In addition to global changes in peripheral WBC counts, we also observe changes within individual WBC populations. Specifically, at 4 days post-infection, LD-fed male mice WBC populations have a significantly lower proportion of lymphocytes than all other groups and a significantly higher proportion of monocytes than ND-fed mice (Fig. 3C). A similar, though not significant, phenotype is observed again at 21 days post-infection, with LD-fed male mice WBC populations again having a lower proportion of lymphocytes and higher proportion of monocytes than all other groups (Fig. 3E). In general, the LD diet seems to promote this phenotype as both LD diet-fed males and females have significantly lower proportions of lymphocytes and significantly higher proportions of monocytes than their ND-fed counterparts at 4 and 21 days post-infection (Fig. 3C and E). Interestingly, in between these time points, WBC populations seem to normalize between groups with no significant differences in WBC populations between relevant groups (Fig. 3D).

### Influence of diet prior to *Salmonella* infection on systemic cytokine production

**Fig 4:**
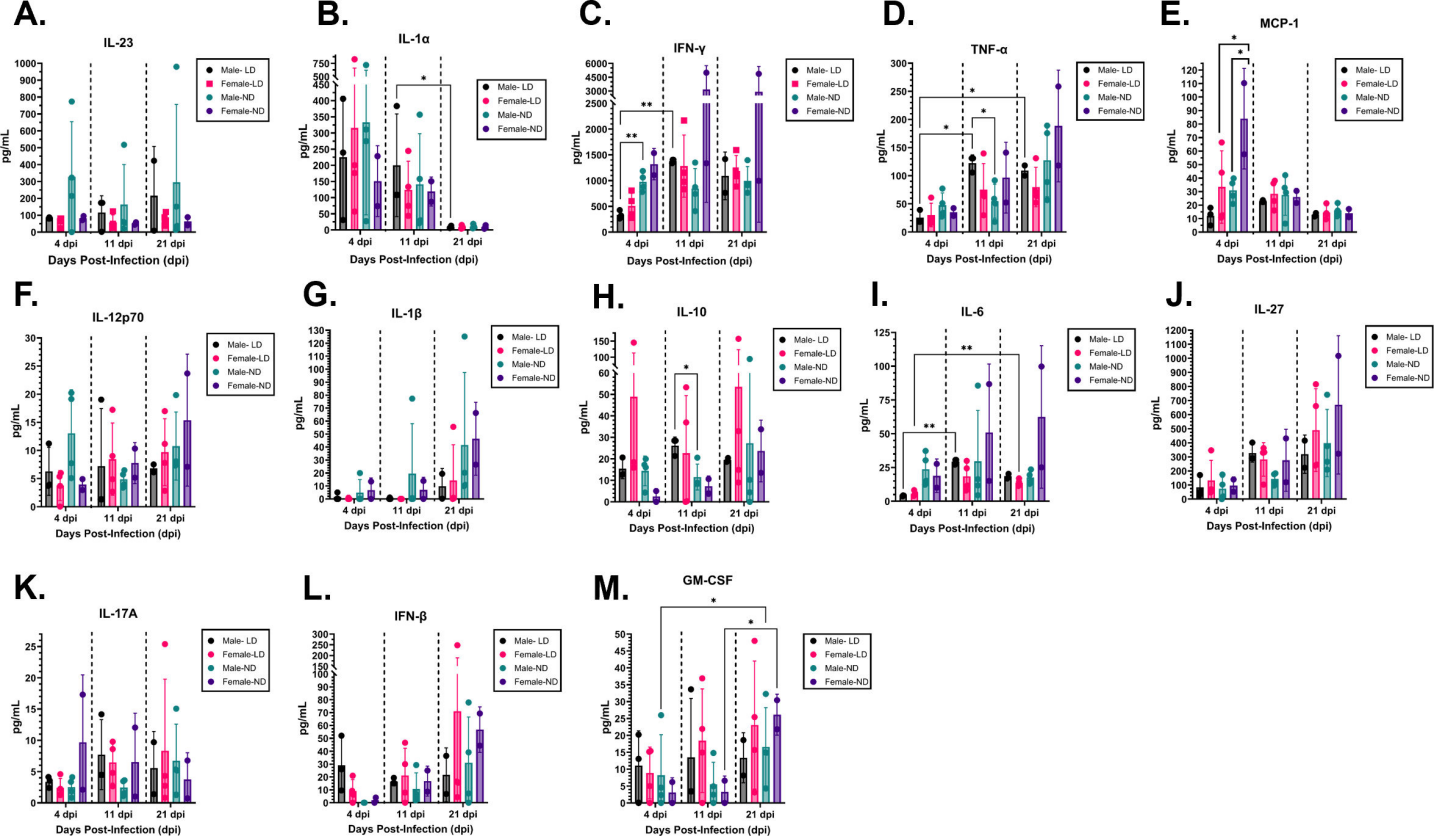
Diet contributes to limited differences in cytokine production following *Salmonella* infection. Using the LEGENDplex Mouse Inflammation panel, serum concentrations of (**A**) IL-23, (**B**) IL-1α, (**C**) IFN-γ, (**D**) TNF-α, (**E**) MCP-1, (**F**) IL-12p70, (**G**) IL-1β, (**H**) IL-10, (**I**) IL-6, (**J**) IL-27, (**K**) IL-17A, (**L**) IFN-β, and (**M**) GM-CSF of the mice shown in Fig. 3 were measured. Graphs represent average and standard deviation of cytokine (pg/mL) concentration per group (*n* = 2–4), with symbols representing individual subjects. Cytokine concentrations are compared between diet-sex cohorts within the same day post-infection as well as individual diet-sex cohorts between days post-infection, using mixed-effects analysis with Tukey’s multiple comparisons test. *P < 0.05; **P < 0.01; all other comparisons are not significant.

In humans, cytokines and chemokines are important mediators of the response to *Salmonella* infection, and deficiencies in specific cytokines, including IFN-γ, IL-12, and IL-23, result in increased susceptibility to *Salmonella* infections (36, 42–44). Given the evidence that *Salmonella* directly influences inflammation and cytokine production in mice (19, 26, 28, 29, 45–47), we wanted to next investigate if infection prompts a different peripheral cytokine response in male mice in comparison to female mice. We wanted to explore a diverse number of inflammatory molecules; however, due to the limited serum volume, we used the LEGENDplex Mouse Inflammation panel. This panel utilizes fluorescent multiplex beads measured with a flow cytometer to simultaneously quantify multiple cytokines in the same serum sample. Using serum collected from the mice shown in Fig. 3, we measured the serum concentration of IL-1α, IL-1β, IL-6, IL-10, IL-12p70, IL-17A, IL-23, IL-27, CCL2 (MCP-1), IFN-β, IFN-γ, TNF-α, and GM-CSF on days 4, 11, and 21 post-infection and compared cytokine levels between LD- and ND-fed male and female mice (Fig. 4).

Following *Salmonella* infection, there were many differences in trends over 4–21 days post-infection within individual groups. Significant increases in IFN-γ, TNF-α, and IL-4 between day 4 and day 11 post-infection and in TNF-α between days 4 and 21 post-infection were observed in LD-fed male mice but not any other groups (Fig. 4C, D and I), although a significant decrease in IL-1α between day 11 and day 21 post-infection was observed in LD-fed male mice (Fig. 4B). A significant increase in IL-4 between days 4 and 21 post-infection was observed only in LD-fed female mice (Fig. 4I). Additionally, we observed a significant increase in GM-CSF between day 4 and day 11 in ND-fed male mice and day 11 and day 21 post-infection in ND-fed female mice. We also examined differences between groups within the same day post-infection. On day 4 post-infection, we observed significantly higher levels of IFN-γ in ND-fed male mice in comparison to LD-fed male mice (Fig. 4C). In addition, on day 4 post-infection, ND-fed female mice produced significantly more MCP-1 than LD-fed females or ND-fed males (Fig. 4E). On day 11 post-infection, LD-fed males produced significantly more TNF-α and IL-10 than ND-fed males (Fig. 4D and H). Thus, while there were some specific differences in the highly *Salmonella*-susceptible LD-fed male mice, no clear patterns emerged related to diet alone.

### Systemic cytokine production following *Salmonella* infection differs between male and female mice

**Fig 5:**
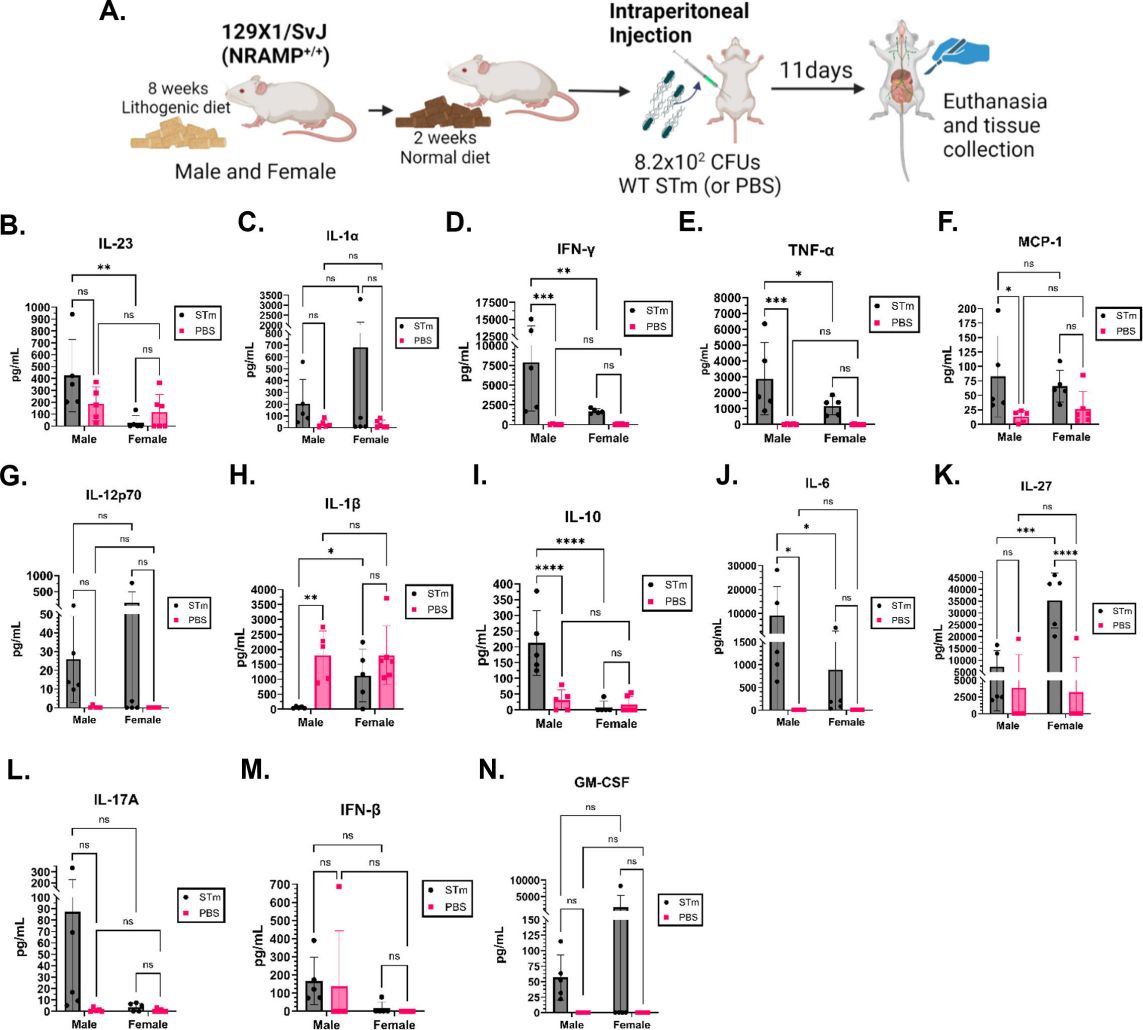
Male mice exhibit higher levels of pro-inflammatory cytokines in response to *Salmonella* infection. (**A**) Male and female mice were fed a lithogenic diet for 8 weeks followed by a normal diet for 2 weeks. Mice were then infected i.p. with 8.2 × 10^2^ CFUs of WT *S*. Typhimurium (STm) or an equal volume of sterile phosphate buffered saline (PBS). Mice were euthanized at 11 days post-infection. Created with Biorender.com. (**B–N**) Serum from infected mice and PBS control mice were used to measure (**B**) IL-23, (**C**) IL-1α, (**D**) IFN-γ, (**E**) TNF-α, (**F**) MCP-1, (**G**) IL-12p70, (**H**) IL-1β, (**I**) IL-10, (**J**) IL-6, (**K**) IL-27, (**L**) IL-17A, (**M**) IFN-β, and (**N**) GM-CSF, using the LEGENDplex Mouse Inflammation panel. Graphs represent average and standard deviation of cytokine (pg/mL) concentration per group (*n* = 5–6), with symbols representing individual subjects. Cytokine concentrations are compared between groups, as indicated, using a two-way ANOVA with uncorrected Fisher’s LSD test. ns = not significant; *P < 0.05; **P < 0.01; ***P < 0.001; ****P < 0.0001.

While we have established that diet induces minor changes in immune response to *Salmonella* infection, we next wanted to determine if sex influences cytokine production in this mouse model independent of infection and following *Salmonella* infection. To compare the baseline immune status to the systemic response to *Salmonella* infection, we measured 13 individual cytokine levels in the serum of the lithogenic diet-fed infected mice and age-matched control mice injected with sterile PBS as a control. We fed male and female 129X1/SvJ mice a lithogenic diet for 8 weeks, followed by 2 weeks on normal diet. Mice were then infected i.p. with 8.2 × 10^2^ CFUs of WT *S*. Typhimurium 14028 (STm) and monitored for 11 days (Fig. 5A). At 11 days post-infection, we euthanized all mice and collected their gallbladder, liver, spleen, cecum, peripheral blood, and serum for CFU enumeration and cytokine quantification. As expected, none of the mice died before being collected at 11 days post-infection; however, male mice had significantly higher bacterial burden than females in all organs (Fig. S4a through e), further supporting our findings that males less effectively control *Salmonella* infections. Importantly, data from Fig. 3 show that WBC counts and populations at 11 days post-infection are similar between males and females, so we can identify differences in cytokine levels, independent of systemic WBC levels.

In all 13 cytokines tested, control males and female mice had no differences in serum cytokine levels (Fig. 5B through N). This suggests that at baseline, male and female mice in our model do not have different systemic inflammatory profiles and that subsequent differences in inflammatory profiles are likely a direct response to infection. STm-infected females produce significantly higher amounts of IL-27 than their PBS control counterparts (Fig. 5K), while STm-infected males produce more IFN-γ, TNF-α, MCP-1, IL-10, and IL-6, but less IL-1β than their PBS control counterparts (Fig. 5B, D through F and H through J). In comparison to STm-infected females, STm-infected males produce significantly more IL-23, IFN-γ, TNF-α, IL-10, and IL-6 and significantly less IL-1β and IL-27 (Fig. 5B, D, E and H through J).

We chose to validate our findings in the multiplex assay with single-cytokine ELISAs testing serum concentrations of IFN-γ, TNF-α, and IL-10. Similar to the results of the multiplex assay, we measured significantly higher levels of IFN-γ and TNF-α in infected male mice by comparison to infected female mice or PBS control male mice (Fig. S4f and g). Notably, the ELISAs showed that infected female mice had lower levels of TNF-α and IL-10 than PBS control females, and no other differences between any other groups were observed in TNF-α and IL-10 levels (Fig. S4g and h). This differs from findings in the multiplex assay, but this may be explained by the lower numbers of mice used in the ELISAs because of the limited serum volume. Together, this suggests that female mice mount a less severe inflammatory response to *Salmonella* infection and that this may contribute to better bacterial control and lower mortality.

## DISCUSSION

The results of this study support the hypothesis that the systemic response to *Salmonella* infection in male 129X1/SvJ mice diverges from that of female mice toward a less protective state associated with higher mortality. We identified differences in survival and bacterial load in male and female mice and examined their connection to systemic inflammation. To facilitate acute, systemic *Salmonella* infection and the transition to chronic infection typified by *Salmonella* biofilm production on gallstones, we employed a mouse strain (129X1/SvJ) with reduced susceptibility that was fed a lithogenic diet. Utilizing *in vivo* bioluminescent imaging, we observed that within 3 days, male mice had a much higher bacterial burden than female mice, and this correlated with males having significantly reduced survival than female mice. We then measured the bacterial burden in mice infected with a lower infectious dose. This did not result in lethal infection but did recapitulate the increased bacterial load phenotype, suggesting that while mortality does depend on the infectious dose, the sexual dimorphism in bacterial load holds true regardless of the infectious dose.

This finding provided an excellent opportunity to compare the systemic response between male and female mice to *Salmonella* infection in the presence and absence of pre-existing cholecystitis. Because these mice appeared otherwise healthy, we could use them to identify any changes in inflammation that may contribute to increased mortality in male mice when given higher infectious doses. We found that WBC populations in serum differ significantly during the acute and chronic phases of infection in male and female mice. This phenotype is characterized by an expansion of the monocytic population and a contraction of the lymphocytic population in male mice. Notably, this observation (either the expansion or contraction) may be related to the observed increased mortality in males during the acute phase of infection, necessitating further experimentation. Additionally, while WBC populations normalize at 11 days post-infection, this does not correlate with normalization of cytokine production between groups, suggesting functional differences in males and females between the same WBC populations. In general, we found that infected male mice largely present with a greater and more diverse immune response than infected female mice. In response to *Salmonella*, male mice produced more canonically pro-inflammatory cytokines, specifically IL-23, IFN-γ, and TNF-α, than female mice. IL-23, IFN-γ, and TNF-α production is known to have a protective role in response to gastrointestinal *Salmonella* infection by activating members of the innate immune system (35, 48–50). Of note, excessively high levels of IFN-γ and TNF-α are associated with sepsis and endotoxic shock in mice (50–52), though those observed here were not at these levels.

Interestingly, IL-1β, a pro-inflammatory cytokine that increases endothelial cell permeability to allow neutrophils to infiltrate the gastrointestinal tract and other infected tissues (53), was decreased in infected males in comparison to uninfected males or infected females. In gastroenteritis models of *Salmonella* infection, it has been shown that IL-1β production is detrimental to host survival (46, 54, 55), so it is surprising that in our typhoidal model, males, but not females, showed decreased IL-1β levels in response to infection. Female mice showed increased IL-27 production in response to infection, which is to be expected given that IL-27 is known to enhance protective responses to *Salmonella* infection (56). Male mice have significantly lower levels of IL-27 than infected females and fail to increase IL-27 levels above that of PBS control males. This could contribute to the male’s reduced ability to control *Salmonella* infection. Interestingly, infected male mice also have increased levels of MCP-1, a chemokine also known as CCL2, which is known to recruit monocytes and memory T cells to sites of infection (57–59). This could reflect some effort to compensate for a poor protective response to *Salmonella* infection and may contribute to tissue damage.

We demonstrated that infected male mice produce more IL-10 and IL-6 than infected females or PBS control males. While IL-10 production often has an anti-inflammatory and protective role in other models of infection, it is well known to be detrimental to the host in animal models of *Salmonella* infection (50, 55, 60). Specifically, IL-10 production from lymphoid cells dampens macrophage and T-cell responses to *S*. Typhimurium and supports bacterial dissemination (50, 61–64). Given that males produce high levels of IL-10 in response to infection, this could contribute to the higher bacterial burdens in males and eventual increase in mortality in male mice given a higher infectious dose. This would also explain why females are better able to control the bacterial burden as their lower levels of IL-10 may prevent inhibition of macrophage and T-cell activity, which are essential to protection from *Salmonella* infection (61, 65, 66). The role of IL-6 in *Salmonella* infection is less clear, with some sources suggesting a pro-inflammatory and detrimental effect in infection (50, 51) while others suggest an anti-inflammatory and protective effect in infection (55, 67, 68).

An important consideration for our conclusions is that mice were fed a lithogenic diet, resulting in cholelithiasis and chronic gallbladder inflammation, prior to the *Salmonella-*induced injury and systemic inflammatory response. Given that cholelithiasis in the absence of infection is associated with inflammation and dysplastic changes in the gallbladder (69), we questioned how cholecystitis may influence the differing response to infection between male and female mice. We addressed this question by feeding mice of both sexes either a lithogenic or normal diet, and following infection, we measured organ bacterial burden and the overall probability of survival. Consistent with previous results, lithogenic diet-fed infected males had a lower probability of survival than lithogenic diet-fed females or uninfected males. No differences in survival were observed between any other diet-fed groups, suggesting that survival is not related to diet. Supporting recent findings (22), we observed that lithogenic diet supports higher cecum colonization in male mice.

Local tissue injury and inflammation in *Salmonella* infection induce systemic responses through cytokine signaling and leukocyte recruitment. *Salmonella* invasion of normal gallbladder epithelial cells in susceptible mouse strains causes significant damage and triggers secretion of pro-inflammatory cytokines and chemokines that recruit neutrophils to the site of infection (70). The literature suggests that pre-existing cholecystitis appears to prolong this response as the majority of studies report that chronically infected resistant mice with gallstones have higher gallbladder *Salmonella* carriage and infiltrating neutrophils (20, 71). We did not observe increased gallbladder carriage in lithogenic diet-fed mice; however, this may be a consequence of low bacterial numbers observed in these experiments rather than a divergence from what is seen in the literature. With this in mind, we investigated how the lithogenic diet and its resulting inflammation influences systemic inflammation following *Salmonella* infection. In mice fed a normal diet prior to infection, there are similar cytokine responses over time to *Salmonella* infection to mice fed a lithogenic diet in both sexes. This includes significant increases in IFN-γ and TNF-α. Uniquely, at early infection time points, the normal diet-fed male mice produce significantly more IFN-γ and significantly less IL-10 and TNF-α than lithogenic diet-fed male mice. GM-CSF is released by monocytes and macrophages in response to *Salmonella* exposure (60, 72). Increased levels of GM-CSF in infected male mice by comparison to infected female mice may represent an effort to compensate for an otherwise inadequate response to infection.

The roles of in IL-1α in male mice fed a normal diet are less clear. In response to *Salmonella* infection, IL-1α levels are known to increase in both sensitive and resistant mice (73); however, increased IL-1α only results in increased survival if administered prior to *Salmonella* infection, not after infection (74, 75), calling into doubt its protective quality during infection. Collectively, these results support that the diet prior to *Salmonella* is associated with differences in peripheral blood WBC counts and systemic cytokine secretion in response to infection, but these changes do not seem to influence the mortality. Future research is needed to determine the relationship of organ-specific inflammatory response and pathologic changes to the systemic responses identified in this study.

In summary, we report that male and female mice of a strain with reduced susceptibility to *Salmonella* infection do not respond similarly to infection. Male mice exhibit reduced survival and increased bacterial burden after infection. In addition, systemic cytokine levels are altered in male mice by comparison to female mice, suggesting males mount a poor protective response to *Salmonella* infection. This finding is important given that in humans, males have higher rates of morbidity with acute *Salmonella* infection, as well as generally providing broader evidence of sex-based differences in infectious disease. Currently, no mechanism is known for this sex-based difference in *Salmonella* infection in humans; however, our study using male and female 129X1/SvJ mice provides important insights into how systemic cytokine levels may contribute to this phenotype.

## Data Availability

Additional supporting data sets may be accessed at 10.5061/dryad.95x69p8z9

## SUPPLEMENTAL MATERIAL

Supplemental material iai.00028-26-s0001.docx Fig. S1 to S4.

ASM does not own the copyrights to Supplemental Material that may be linked to, or accessed through, an article. The authors have granted ASM a non-exclusive, world-wide license to publish the Supplemental Material files. Please contact the corresponding author directly for reuse.
