# Supplementary material for: Enhanced Salmonella mortality in naturally resistant, immunocompetent male mice is associated with an altered systemic immune response
Source: Infect Immun. 2026 Mar 11;94(4):e00028-26. doi: 10.1128/iai.00028-26 (PMC13081722; doi:10.1128/iai.00028-26)

**Supplemental Materials**

**Enhanced *Salmonella* mortality in naturally resistant, immunocompetent male mice is associated with an altered systemic immune response**

Aliyah N. Bennett^1,2^, Allysa L. Cole^1, 2, 3^, and John S. Gunn^1,2,4*^

^1^Center for Microbe & Immunity Research, Abigail Wexner Research Institute at Nationwide Children's Hospital, Columbus, OH, USA

^2^Infectious Diseases Institute, The Ohio State University, Columbus, Ohio, USA.

^3^Department of Veterinary Biosciences, The Ohio State University, Columbus, Ohio, USA

^4^Department of Pediatrics, College of Medicine, The Ohio State University, Columbus, Ohio, USA


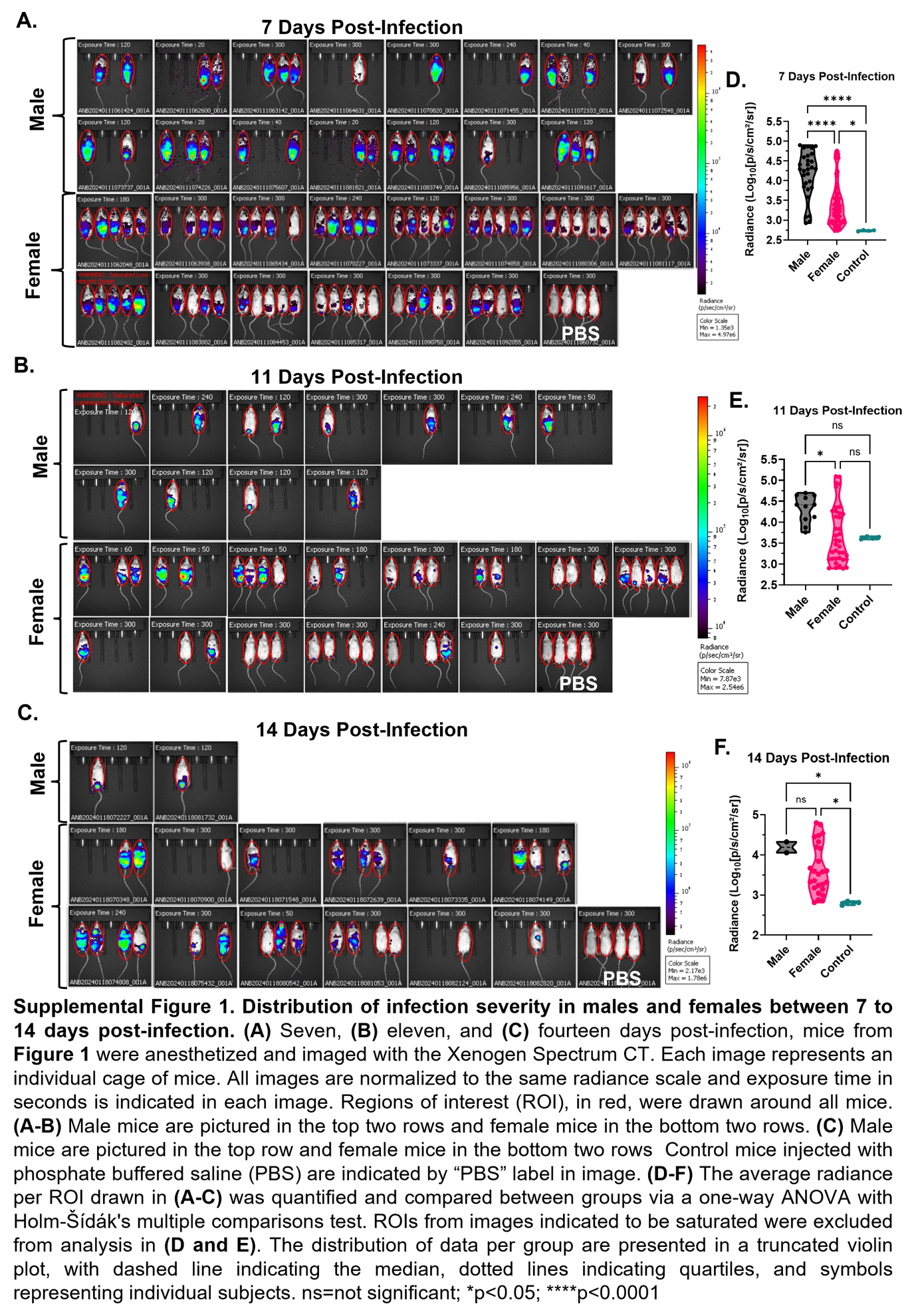


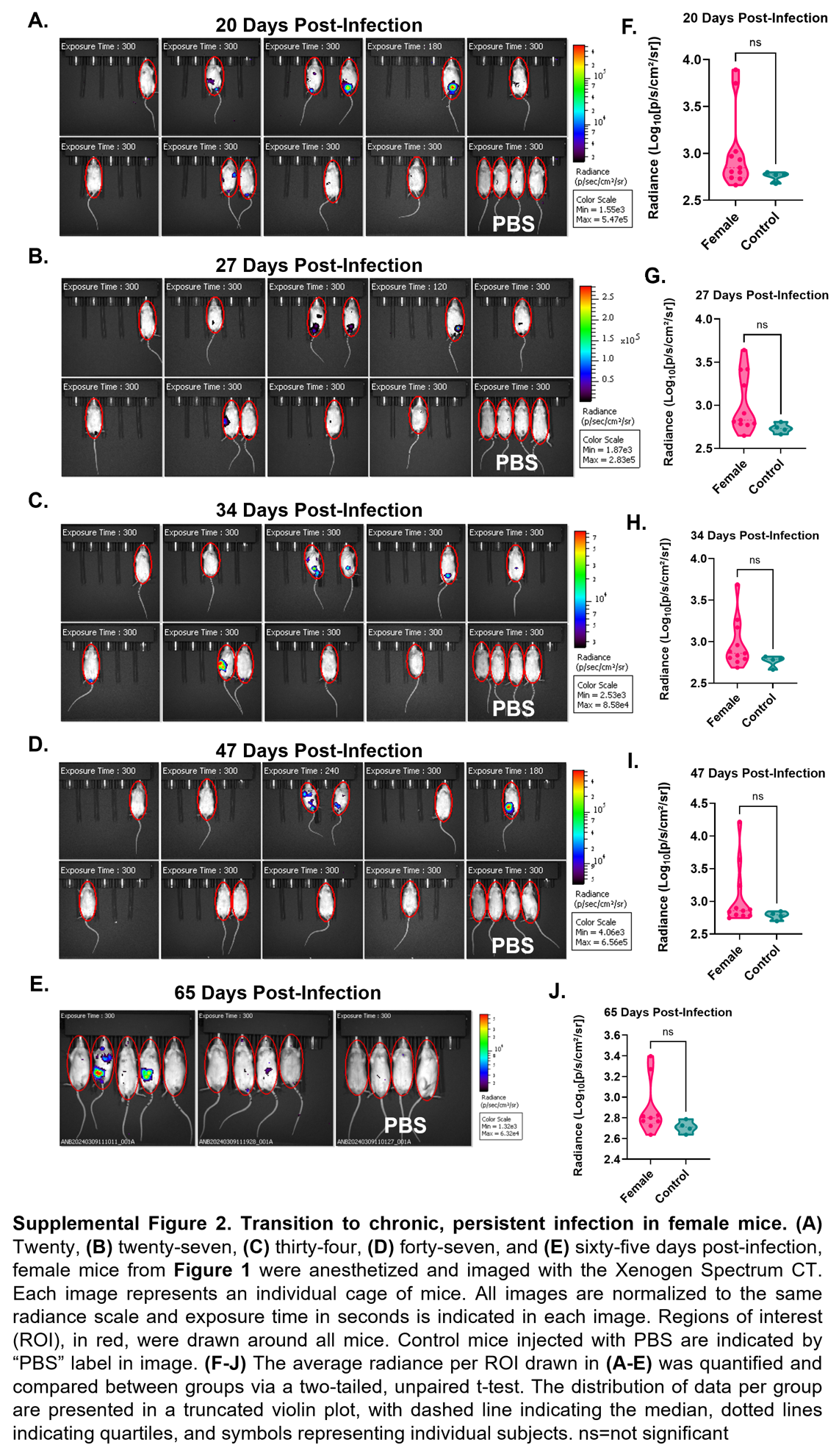


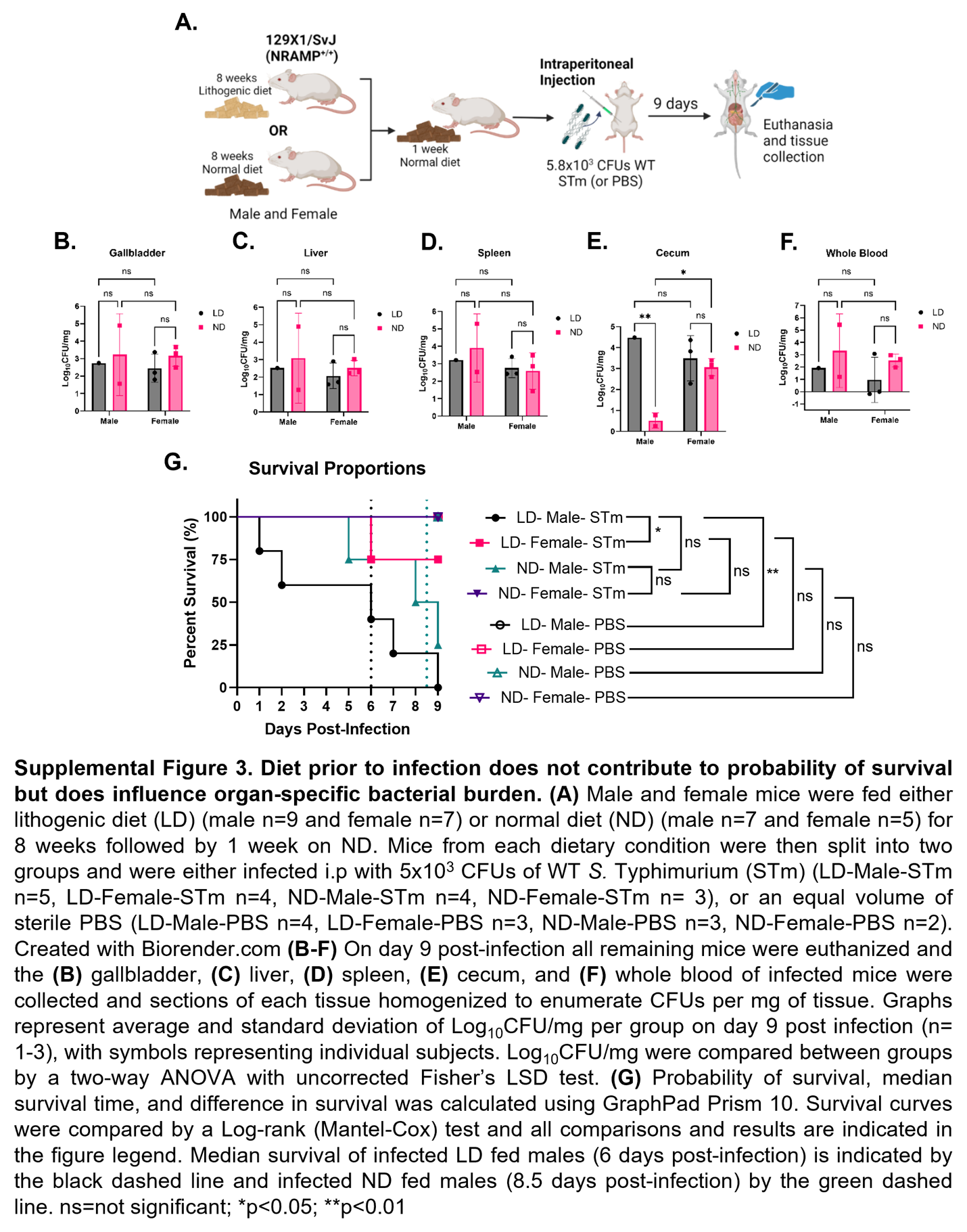


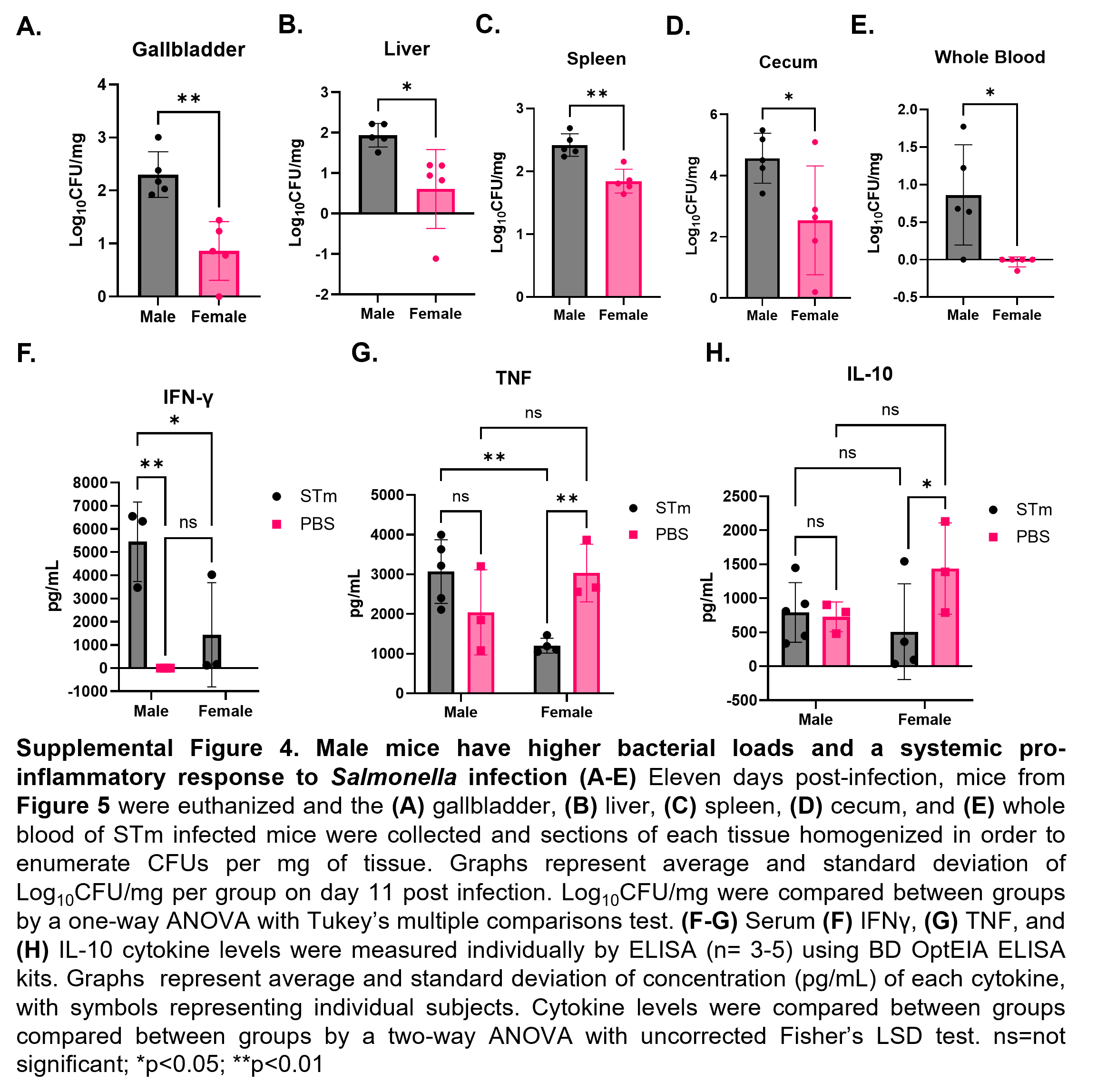

Supplement: Supplemental material — Fig. S1 to S4. [file iai.00028-26-s0001.docx]
